# Tom Johnson, of Cleveland

**Published:** 1901-06

**Authors:** 


### TOM JOHNSON, OF CLEVELAND.

FROM the Cleveland *Plain Dealer*, it is learned that Mayor Johnson places himself on record as not believing in vaccination, and does not believe in "contaminating a man's blood with poison;" that "no doctor would pump the virus into me." He also announces that he will not approve of general or compulsory vaccination. The effect of such rash statements and his attitude toward the present epidemic now prevailing in his city, cannot but be far reaching in its possibility, both on account of his personality and his official position, inasmuch as there are very many who do not understand the reason for, or justification of, sanitary laws and regulations. They see no reason why what was good enough for their fathers and grandfathers before them should not be good enough for them.

When it is considered what vaccination has done for the human race; what the present consensus of opinion of the scientific and medical world is concerning it, it is simply with astonishment that we learn the fact that he is opposed to the progress of civilisation, and much that is conducive to the material and moral welfare of the masses. Mr. Johnson is, without doubt, an aggressive business man, an astute politician, and a strong personality; hence it intensifies our astonishment that he should take such a position and announce such views without first becoming acquainted with the facts, and, more particularly, as it is regarding a matter about which he can have no personal knowledge and surely cannot be informed without much study of the subject.

Were he conversant with the facts, he would surely hesitate long in placing his opinion over against that of unselfish and self-sacrificing medical men and the evidence of history. He assuredly cannot be impressed sufficiently with the fact that this is a terrible thing to be believed. Loose statements about poison contaminating a man, and the like, are much to be regretted. Were he familiar with the simple fact that vaccination does modify or prevent the disease, *i. e.*, has, does, and will check the spread of the malady; and, further, that in his city, according to the reports of the United States Government, from 84 cases on January 18, 1901, the disease has increased to 845 cases by May 17, 1901, which, only in part, represents the number of existing cases, many being concealed and not reported, and had he, upon his inauguration into office, recognised and enforced the methods of sanitary science, Cleveland would not have to its discredit a pestilence that is increasing in extent as shown by the United States Marine Hospital Service reports. This increase has been largely from non-enforcing of protection by vaccination, which dereliction is as inhuman as it is unsanitary.

The Encampment of the Grand Army of the Republic is to be held at Cleveland this summer. It is difficult to suppress something like indignation at his failure to avail himself of and approve every method, and the most important one, in checking this spreading pestilence, and particularly in view of the approaching influx of elderly men—veterans. Every death, from this disease, that has occurred, has been preventable; every new death or case in the absence of vaccination is also preventable; every individual visiting Cleveland and contracting the disease or who may carry the infection away with him to others is unnecessary, and who is responsible? Reflect, Mr. Johnson !

Mayor Johnson, the objections made to vaccination, and the controversies with regard to its efficacy, are matters of common knowledge, but the general result of all scientific inquiries has been to confirm its protective powers. It is a safe and comprehensive procedure. It is in the direction of improved sanitation and an enlightened municipal administration. You cannot afford to commit an act inconsistent with the maintenance of the public health. Educate yourself, in order that you may be capable of comprehending and appreciating its true significance. Do not adopt the professional style of the "antis" by expressing opinion in the daily press suited to the level of the ignorant. Try it, become convinced and see for yourself how quickly and happily it will banish the smallpox pestilence from the shores of your city—now a center of infection that has attracted national attention.

Your extreme ignorance concerning the whole matter, or rather your pretence of knowledge of what is good for the good of the community is to be deprecated. Do not be hostile to the attainment of the truth. Change your false, absurd and dangerous notions; without a change, there can be no advance; do not listen to the ridiculous promises of charlatans, or entertain the injurious influences of the antivaccinationists.

You are an announced reformer—reform! Be advised by your leading medical men who have the courage to advocate and practice their art conscientiously, however opposed by vulgar prepositions and prejudices. Vaccinate! Don't turn your back to the light, but check the development and spread of this most loathsome epidemic by availing yourself of the most important of measures—vaccination.

---

GOVERNOR ODELL has vetoed all the bills passed by the last legislature granting special privileges to certain persons with reference to entrance into the several professions. These bills were passed in the interest of certain individuals who sought to gain entrance into the learned professions without complying with the requirements now fixed by law. Governor Odell is entitled to the thanks not only of every professional man in the state, but of every other citizen for this wise and beneficent action. His vetoes ought to be taken as a rebuke by every member of the legislature who introduced these measures, or who labored to obtain their enactment.

---

JUDGE LUMPKIN, of the Superior Court of Georgia, has denied the application for a charter for "The Atlanta Institute of Christian Science," the effect of his decision being that Christian scientists cannot practice their treatment of diseases in the State of Georgia without having been regularly graduated in medicine or passed an examination before the medical examining board, the same as other physicians. Judge Lumpkin holds that, according to the decision of a case in the Supreme Court of Nebraska, Christian science is the practice of medicine, and he further holds that the practice of medicine in Georgia, according to the state law, must be accomplished by persons who are regularly graduated from a medical school.

---

THE anti-spitting ordinance, which was proposed and pushed to a definite conclusion by Health Commissioner Wende in the face of much opposition, went into effect May 21, 1901. It is a wise measure and will serve to place Buffalo in the first rank with those cities that have adopted similar measures to curtail a filthy and disease-spreading habit.

The *Buffalo Morning Express*, in its issue of May 11, 1901, made the following sensible comments regarding it:

"Now that the mayor has signed the anti-spitting ordinance, it should be strictly enforced. The ordinance prohibits spitting on the floors of street cars and other public conveyances, and on the floors of stores, factories, theaters and public buildings, and prescribes a fine of not less than $2 or more than $100 for each offense. Sidewalks are excepted from the ordinance, but they should have been included. The ordinance will be published for ten days, after which its enforcement will begin.

"Undoubtedly the enforcement will work some hardship, but that is unavoidable when an attempt is made to break a habit of such long standing. The persons before whom the cases are heard will, of course, discriminate between carelessness and contumacy in the matter of fines, but the quickest way to break up the objectionable practice is to permit no offender to escape without a fine."

